# Investigating the relationship between energy expenditure, walking speed and angle of turning in humans

**DOI:** 10.1371/journal.pone.0182333

**Published:** 2017-08-10

**Authors:** M. A. McNarry, R. P. Wilson, M. D. Holton, I. W. Griffiths, K. A. Mackintosh

**Affiliations:** 1 Applied Sports, Technology, Exercise and Medicine Research Centre, College of Engineering, Swansea University, Swansea, United Kingdom; 2 Swansea Lab for Animal Movement, Biosciences, College of Science, Swansea University, Swansea, United Kingdom; 3 Visual Computing, Computer Science, College of Science, Swansea University, Swansea, United Kingdom; Universita degli Studi di Verona, ITALY

## Abstract

Recent studies have suggested that changing direction is associated with significant additional energy expenditure. A failure to account for this additional energy expenditure of turning has significant implications in the design and interpretation of health interventions. The purpose of this study was therefore to investigate the influence of walking speed and angle, and their interaction, on energy expenditure in 20 healthy adults (7 female; 28±7 yrs). On two separate days, participants completed a turning protocol at one of 16 speed- (2.5, 3.5, 4.5, 5.5 km∙h^-1^) and angle (0, 45, 90, 180°) combinations, involving three minute bouts of walking, interspersed by three minutes seated rest. Each condition involved 5 m of straight walking before turning through the pre-determined angle with the speed dictated by a digital, auditory metronome. Tri-axial accelerometry and magnetometry were measured at 60 Hz, in addition to gas exchange on a breath-by-breath basis. Mixed models revealed a significant main effect for speed (F = 121.609, *P* < 0.001) and angle (F = 19.186, *P* < 0.001) on oxygen uptake ($\dot{V}{\text{O}}_{2}$) and a significant interaction between these parameters (F = 4.433, *P* < 0.001). Specifically, as speed increased, $\dot{V}{\text{O}}_{2}$ increased but significant increases in $\dot{V}{\text{O}}_{2}$ relative to straight line walking were only observed for 90° and 180° turns at the two highest speeds (4.5 and 5.5 km∙hr^-1^). These findings therefore highlight the importance of accounting for the quantity and magnitude of turns completed when estimating energy expenditure and have significant implications within both sport and health contexts.

## Introduction

A high body mass index is a major risk factor for the incidence of numerous non-communicable diseases (NCD), such as cardiovascular and kidney diseases, diabetes and some cancers [1–6]. Indeed, obesity has been recognised as a major public health challenge for the 21^st^ century [7], with concerns regarding the health and economic burden, which have led to the identification of global targets to halt the rise in obesity prevalence by 2025 [8, 9]. However, recent figures from the NCD Risk Factor Collaboration, which analysed the aggregated data of 19.2 million participants from 200 countries, suggest that if current, post-2000, trends continue, the probability of meeting these targets is almost zero. These findings highlight the critical need to develop and implement effective interventions for the prevention and treatment of excess body mass [10]. A fundamental principle in the development of such interventions is the assessment of the energy expenditure associated with activities of daily living; weight management strategies are most effective when individuals can accurately determine how much energy they have expended [11]. Indeed, a failure to assess energy expenditure appropriately may, at least in part, explain the inconsistent evidence regarding intervention effectiveness and sustainability.

Walking represents a popular, convenient and relatively safe form of activity that can easily be incorporated into weight management programmes [12–14]. The energy expenditure associated with walking are reported to be either linearly or slightly exponentially related to speed [15]. However, the applicability of these findings is based on walking in a straight line which does not align with everyday activities. In particular, recent studies have suggested that the process of changing direction is associated with significant additional energy expenditure [16–18]. Wilson et al. [18] reported that the energy expenditure of turning is linearly related to the degree of turning angle at 6 km∙hr^-1^ while Hatamoto et al. found quadratic [17] or linear [16] functions best represented the relationship between running speed and the energy expenditure of a 180° turn. A failure to account for this additional energy expenditure of turning has significant implications for both sporting and health contexts. For example, most English Premier league footballers make more than 700 turns per match [19] while medical treatment effectiveness is often assessed using a six-minute walking test. While the latter is intended to be conducted over a standardised 30 m straight line distance [20], some studies have used 20 m or 50 m straights [21, 22], significantly influencing the number of turns completed and thus potentially confounding inter-study comparisons. Indeed, the difference in the number of turns completed, and thus overall energy expenditure, may explain studies, which utilised shorter straights reporting significantly shorter distances covered [23–25].

The purpose of the present study was therefore to investigate the influence of walking speed, angle, and their interaction, on energy expenditure. We hypothesised that 1) as walking speed increased, so would energy expenditure; 2) as angle increased, so would energy expenditure and that 3) angle and speed would demonstrate a synergistic effect on energy expenditure while walking.

## Materials and methods

### Participants

In total, 20 healthy adults (7 female, 13 male; 28 ± 7 yrs; 20.5 ± 4.1 kg∙m^2^) were recruited for the study. The participants were all recreationally active but none were highly trained. Prior to testing, participants were informed of the protocol and risks and provided written consent. All procedures were approved by a Swansea University ethics committee and were conducted in accordance with the Declaration of Helsinki. Participants were asked to arrive at the laboratory in a rested state, at least two hours postprandial and to avoid strenuous exercise in the 24 hours preceding each testing session. Participants were also asked to refrain from caffeine and alcohol for 6 and 24 h before each test, respectively. All tests were performed at the same time of day (± 2 h).

### Experimental design

Participants were required to visit the laboratory/indoor track on three occasions, separated by at least 24 hours recovery. Participants initially completed an incremental treadmill test for determination of peak oxygen uptake ($\dot{V}{\text{O}}_{2}{}_{\text{peak}}$) and the gas exchange threshold (GET). On each of the two subsequent visits, participants completed the turning protocol.

### Incremental treadmill test

Following a three-minute warm-up at 6 km·hr^-1^, the treadmill speed increased by 1km·hr^-1^ every minute until participants reached volitional exhaustion. The treadmill gradient was set at 1% throughout the test [26], until participants reached their maximal running speed at which point it subsequently increased by 1% every minute until volitional exhaustion. Participants were given strong verbal encouragement throughout the test.

### Turning protocol

**Fig 1:**
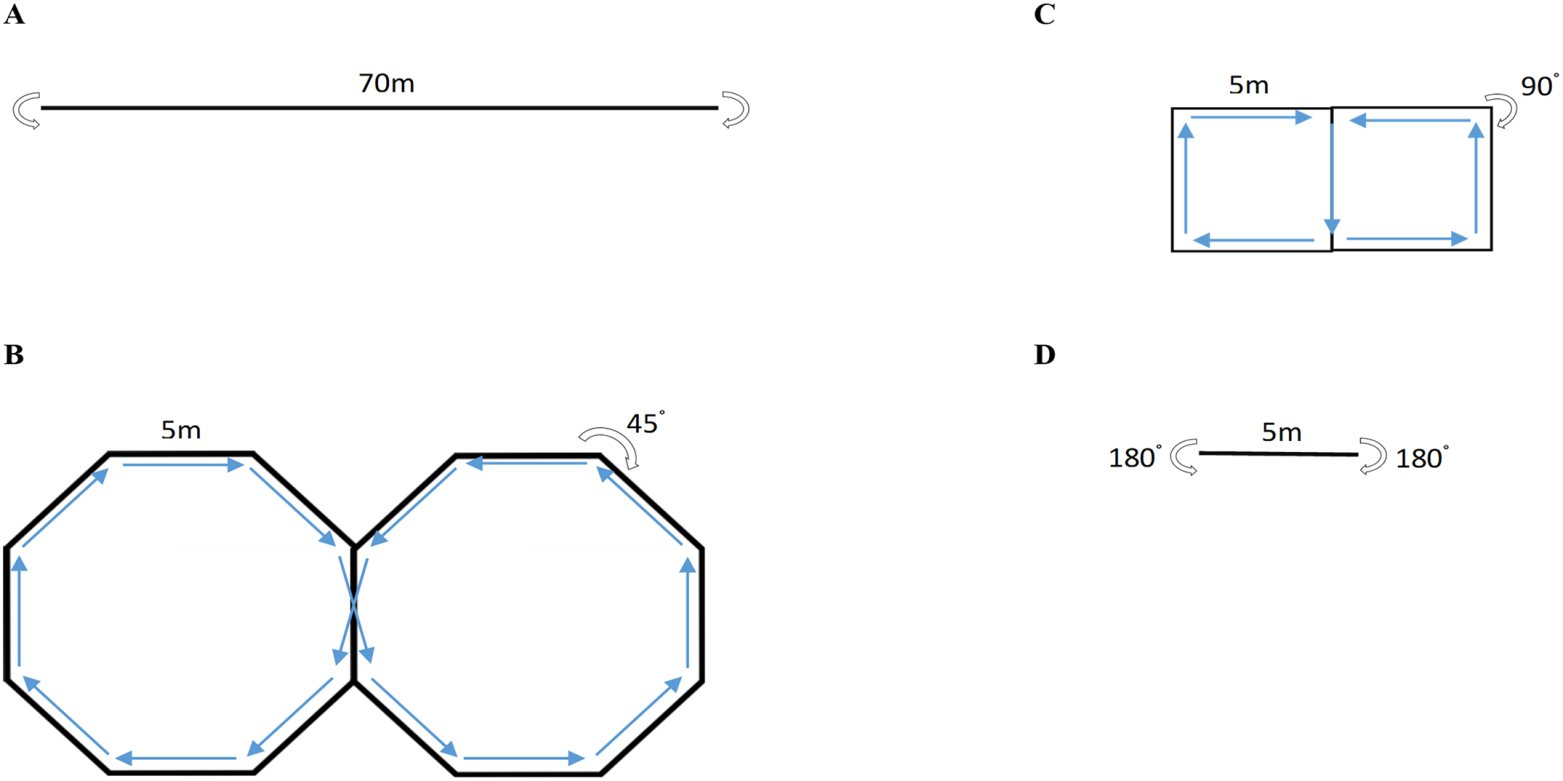
Schematic of the turning protocol showing 5m straights interspersed by prescribed turns of a) 0° b) 45° c) 90° and d) 180° with equal left and right hand turns.

On subsequent visits to the indoor track, each participant was asked to complete repeated three-minute bouts of walking interspersed by three minutes of rest. In a randomised order, each participant walked at four different walking velocities (2.5, 3.5, 4.5, 5.5 km·hr^-1^) in combination with four different angles (0, 45, 90, 180°). Specifically, each of the sixteen conditions involved 5 m of straight walking interspaced by prescribed turns with the speed dictated by a digital, auditory metronome, which sounded once half-way between turns and once on the turns. Each condition incorporated an equal number of left- and right-handed turns, as illustrated in Fig 1.

### Measurements

Throughout all the tests, gas exchange variables (MetaMax Cortex 3B, CORTEX Biophysik GmbH, Germany) were measured on a breath-by-breath basis and displayed online. Prior to each test, the gas analysers were calibrated using gases of known concentration and the turbine volume transducer was calibrated using a 3-litre syringe (Hans Rudolph, Kansas City, MO). The delay in the capillary gas transit and analyser rise time were accounted for relative to the volume signal, thereby time-aligning the concentration and volume signals. Additionally, two combined tri-axial accelerometers and tri-axial magnetometers (SLAM Tracker, Wildbyte Technologies Ltd, Swansea, UK), measuring at 60 Hz, were worn by participants; one set was worn on the right mid-axilla line at the level of the iliac crest and one set at the middle of the lower back.

### Data analysis

The peak $\dot{V}{\text{O}}_{2}$ was defined as the highest 10 s stationary average during the incremental treadmill test. The GET was determined by the V-slope method [27] as the point at which carbon dioxide production began to increase disproportionately to $\dot{V}{\text{O}}_{2}$, as identified using purpose written software developed using LabVIEW (National Instruments, Newbury, UK). The mean $\dot{V}{\text{O}}_{2}$ during each condition was taken as the first 45 seconds of the final minute of that bout. Subsequent analyses were based on the premise that the energy expenditure of turning was superimposed on the baseline energy expenditure of straight line travel. Thus, the difference in $\dot{V}{\text{O}}_{2}$ during straight line walking (0°) at each speed compared to the $\dot{V}{\text{O}}_{2}$ engendered during walking with 45, 90 or 180° turns, was attributed to the additional energy expenditure of turning. This $\dot{V}{\text{O}}_{2}$ was converted to gross energy expenditure in kJ using a conversion factor of 20.1 J per ml of oxygen and subsequently divided by the total number of turns per condition to provide an estimate of the energy expenditure of each angle and speed combination.

The raw accelerometer data were converted to dynamic body acceleration (DBA) by first smoothing each channel to derive the static acceleration using a running mean over 2 s [28] and then subtracting this static acceleration from the raw data [29]. The resulting values for dynamic acceleration were all then converted to positive values. These values for DBA were then vectorially summed to give:

$$VeDBA=\;\sqrt{\left(A_{x}^{2}+A_{y}^{2}+A_{z}^{2}\right)}$$

Where VeDBA is the vectorial dynamic body acceleration, A_x_, A_y_, and A_z_ are the derived dynamic accelerations at any point in time corresponding to the three orthogonal axes of the accelerometer [30].

Mean and summed VeDBA were derived for each individual turn and straight during the middle minute of the each condition and for the overall three minute bout. Individual turns and straights were determined using custom designed C++ software (DDMT Wildbyte Technologies Ltd) written specifically for the SLAM Tracker devices, to visualise the accelerometry and magnetometry traces and identify the point at which each trace significantly deviated from the local mean.

### Statistics

Gaussian distributions in data were confirmed by Shapiro-Wilks tests. To account for the repeated and correlated nature of the data, linear mixed-effects models were used to determine the influence of, and interaction between, walking speed and turn angle on energy expenditure and *VeDBA* (S1 File). All condition combinations were placed in one model with covariates (sex, stature, peak $\dot{V}{\text{O}}_{2}$ and/or turning $\dot{V}{\text{O}}_{2}$ for *VeDBA*) added to subsequent adjusted models to determine their modulatory effect. Pearson product moment correlation coefficients were used to analyse the degree of association between key variables. Statistical analyses were conducted using PASW Statistics 21 (SPSS, Chicago, IL). All data are presented as means ± SD. Statistical significance was accepted when P≤0.05.

## Results

Descriptive characteristics of the sample population are shown in Table 1. The male participants were significantly taller and demonstrated a higher peak $\dot{V}{\text{O}}_{2}$, both in absolute and relative terms (normalised per kg body mass).

**Table 1:** Participant characteristics.

	Total	Male	Female
**n**	10	13	7
**Age, yrs**	28.0 ± 6.7	28.5 ± 7.5	27.2 ± 5.3
**Stature, m**	1.74 ± 0.09	1.78 ± 0.08	1.67 ± 0.02*
**Body mass, kg**	72.1 ± 16.1	74.5 ± 16.1	67.6 ± 16.4
**BMI, kg∙m**^**2**^	20.6 ± 4.1	20.8 ± 3.9	20.3 ± 4.9
**Peak $\dot{V}O_{2}$, l∙min**^**-1**^	3.54 ± 0.75	3.92 ± 0.64	2.85 ± 0.33*
**Relative peak $\dot{V}O_{2}$, l∙kg**^**-1**^**∙min**^**-1**^	49.8 ± 7.9	53.2 ± 5.5	43.5 ± 8.1*
**GET, l∙min**^**-1**^	2.31 ± 0.66	2.52 ± 0.71	1.92 ± 0.34*
**GET, %**	65 ± 9	64 ± 10	67 ± 7

Mean ± SD. BMI, body mass index; $\dot{V}{\text{O}}_{2}$, oxygen uptake, GET, gas exchange threshold.

* Significant difference between males and females P < 0.05

As shown in Table 2, there was a significant main effect for speed (F = 121.609, *P* < 0.001) and turn angle (F = 19.186, *P* < 0.001) on $\dot{V}{\text{O}}_{2}$ and a significant interaction between these parameters (F = 4.433, *P* < 0.001). Specifically, as speed increased, $\dot{V}{\text{O}}_{2}$ increased, but significant increases in $\dot{V}{\text{O}}_{2}$ relative to straight line walking were only observed for 90° and 180° turns at the two highest speeds (4.5 and 5.5 km∙hr^-1^; Table 2). Males demonstrated a significantly greater $\dot{V}{\text{O}}_{2}$ across all conditions (F = 25.322, *P* < 0.001), although this difference was reversed when stature was included in the model (Sex: F = 16.77, *P* < 0.001; Stature: F = 152.493, *P* < 0.001). $\dot{V}{\text{O}}_{2}$ during the turning protocol was dependent on peak $\dot{V}{\text{O}}_{2}$ (F = 100.970, *P* < 0.001) but once scaled to account for differences in body size, this relationship was no longer significant (F = 0.708, *P* > 0.05).

**Table 2:** Mean energy expenditure and *VeDBA* during each combination of walking speed and angle.

	$\dot{V}O_{2}$ (l∙min^-1^)				Mean VeDBA (g)			
	0°	45°	90°	180°	0°	45°	90°	180°
**2.5 km∙hr**^**-1**^	0.66 ± 0.20	0.63 ± 0.19	0.63 ± 0.20	0.67 ± 0.22	0.19 ± 0.03	0.19 ± 0.02	0.19 ± 0.04	0.20 ± 0.03
**3.5 km∙hr**^**-1**^	0.73 ± 0.20*	0.74 ± 0.22*	0.74 ± 0.21*	0.82 ± 0.24*	0.24 ± 0.04*	0.25 ± 0.05*	0.25 ± 0.04*	0.27 ± 0.04*
**4.5 km∙hr**^**-1**^	0.86 ± 0.26*	0.88 ± 0.26*	0.93 ± 0.24*^#^	1.10 ± 0.33*^#^	0.32 ± 0.03*	0.32 ± 0.04*	0.32 ± 0.05*	0.37 ± 0.05*
**5.5 km∙hr**^**-1**^	1.00 ± 0.26*	1.00 ± 0.33*	1.14 ± 0.30*^#^	1.54 ± 0.36*^#^	0.44 ± 0.05*	0.44 ± 0.05*	0.46 ± 0.06*	0.53 ± 0.08*

Mean ± SD. $\dot{V}{\text{O}}_{2}$, net oxygen uptake; *VeDBA*, vectorial dynamic body acceleration.

* indicates significant difference to 2.5 km∙hr^-1^ within angle (*P*<0.05)

^#^ indicates significant difference to straight walking within speed (*P*<0.05)

**Fig 2:**
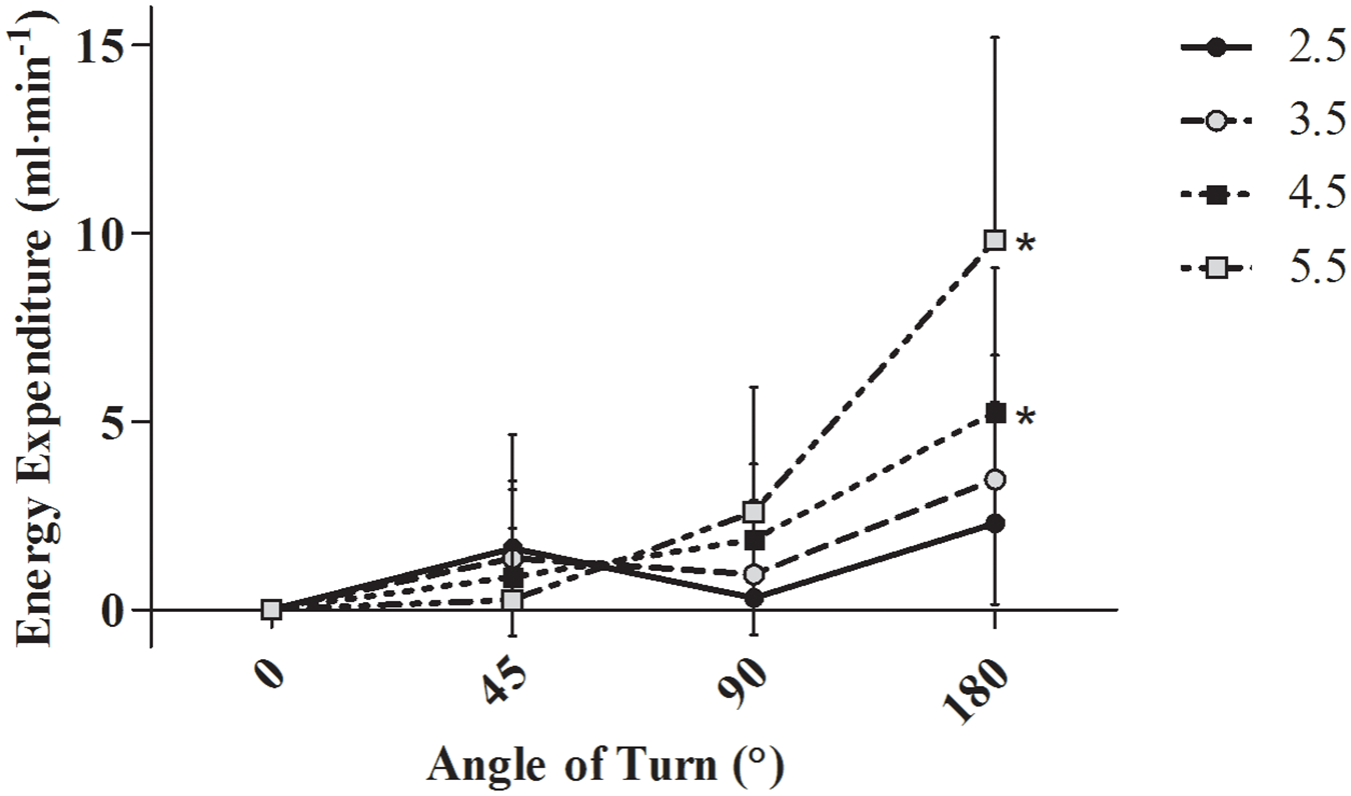
Estimated energetic cost of individual turns at each combination of walking speed and turning angle. * indicates a significant difference in the energy expenditure of turning compared to 45°. Values shown are the difference in energy expenditure between each condition and straight walking.

The estimated energy expenditure associated with an individual turn is represented in Fig 2, showing the synergistic interaction between speed and turn angle in determining the energy expenditure.

In accord with $\dot{V}{\text{O}}_{2}$, there was a significant main effect for speed and angle on mean *VeDBA* during the straights (speed: F = 1058.734, *P* < 0.001; angle: F = 43.416, *P* < 0.001) and corners (speed: F = 996.671, *P* < 0.001; angle: F = 12.534, *P* < 0.001), with a significant interaction effect between speed and angle (straight: F = 4.497, *P* < 0.001; corners: F = 3.053, *P* < 0.01). Specifically, while both straight and corner VeDBA increased with speed, the effect of angle was only manifest at 4.5 and 5.5 km∙hr^-1^ (Table 3). For straight sections, VeDBA was dependent on sex (F = 15.781, *P* < 0.001) and peak $\dot{V}{\text{O}}_{2}$ (F = 7.437, *P* < 0.01) whilst stature was not a significant contributor (F = 0.038, *P* > 0.5). In contrast, corner VeDBA was significantly influenced by sex (F = 6.723, *P* < 0.01) and stature (F = 4.507, *P* < 0.05), but not peak $\dot{V}{\text{O}}_{2}$ (F = 0.243, *P* > 0.05).

**Table 3:** Mean straight and corner *VeDBA* during each combination of walking speed and angle.

	Straight Mean *VeDBA (g)*				Corner Mean *VeDBA (g)*			
	0°	45°	90°	180°	0°	45°	90°	180°
**2.5 km∙hr**^**-1**^	0.19 ± 0.03	0.18 ± 0.03	0.19 ± 0.04	0.20 ± 0.03	0.18 ± 0.04	0.18 ± 0.03	0.18 ± 0.04	0.18 ± 0.03
**3.5 km∙hr**^**-1**^	0.24 ± 0.04*	0.25 ± 0.05*	0.25 ± 0.04*	0.28 ± 0.05*	0.23 ± 0.04*	0.25 ± 0.06*	0.24 ± 0.05*	0.24 ± 0.03*
**4.5 km∙hr**^**-1**^	0.32 ± 0.04*	0.32 ± 0.04*	0.33 ± 0.04*^#^	0.39 ± 0.04*^#^	0.30 ± 0.05*	0.33 ± 0.04*	0.32 ± 0.04*^#^	0.34 ± 0.05*^#^
**5.5 km∙hr**^**-1**^	0.44 ± 0.05*	0.44 ± 0.05*	0.46 ± 0.06*^#^	0.55 ± 0.09*^#^	0.42 ± 0.06*	0.44 ± 0.05*	0.45 ± 0.06*^#^	0.49 ± 0.07*^#^

Mean ± SD. VeDBA, vectorial dynamic body acceleration.

* indicates significant difference to 2.5 km∙hr^-1^ within angle (*P*<0.05)

^#^ indicates significant difference to straight walking within speed (*P*<0.05)

Overall $\dot{V}{\text{O}}_{2}$ during each condition was significantly correlated with straight (r^2^ = 0.61; *P* < 0.001), corner (r^2^ = 0.58; *P* < 0.001) and total mean *VeDBA* (r^2^ = 0.61; *P* < 0.001), demonstrating a weaker but statistically significant relationship with peak $\dot{V}{\text{O}}_{2}$ (r^2^ = 0.36; *P* < 0.001) and stature (r^2^ = 0.32; *P* < 0.001).

## Discussion

This is the first study to investigate the interaction between speed and turn angle in determining the energy expenditure associated with walking. In agreement with one hypothesis, as speed increased for any given turning angle, the associated energy expenditure similarly increased. However, whether angle comprised a significant additional energy expenditure was dependent on the degree of turn angle. Specifically, irrespective of speed, 45° turns did not significantly increase energy expenditure, whilst 180° turns were always associated with a greater energy expenditure than straight line walking. Speed and angle demonstrated a significant interaction; 90° turns were only associated with significantly increased energy expenditure relative to straight line walking at 4.5 and 5.5 km∙hr^-1^. This synergistic interaction was further supported by the exponential relationship found to best represent the relationship between speed and angle [15]. These findings therefore highlight the importance of accounting for the quantity and magnitude of turn completed when estimating energy expenditure, particularly at higher speed and angles.

In recent years there has been increasing recognition of the physiological demands engendered by turning 180° when running. Dellal et al. [31] reported a greater heart rate, blood lactate and ratings of perceived exertion (RPE) during intermittent shuttle runs involving 180° turns compared to straight line running at the same average running velocities, subsequently confirmed by Buchheit et al. [32]. Furthermore, Bekraoui et al. [33] found that covering the same distance at the same average speed resulted in a significantly greater physiological response when the course was 3.5m compared to 7.0m. These earlier findings were recently extended by Hatamoto et al. [17] who found that, even at running speeds as low as 3 km∙hr^-1^, thirty 180° turns per minute elicited a similar metabolic demand as straight line running at 6 km∙hr^-1^. In the present study, a significant increase in total energy expenditure relative to straight line walking was not observed at 2.5 km∙hr^-1^, but was observed at 3.5 km∙hr^-1^. Whilst these findings are largely in accord with those of Hatamoto et al. [17], it is pertinent to note certain methodological discrepancies, such as the training status of the sample population and turning frequencies utilised, which limit inter-study comparisons. Specifically, there were considerable differences in the number of turns completed, with Hatamoto et al. [17] utilising up to 30 turns per minute compared to the 35 turns in 3 minutes at 3.5 km∙hr^-1^ used in the present study.

The greater energy expenditure associated with turning whilst walking is likely to be primarily attributable to the deceleration and subsequent acceleration required to make a turn, both of which necessitate eccentric and concentric muscle contractions, respectively [34]. Acceleration has been shown to engender a greater energy expenditure than travelling at a constant speed, with the energy expenditure dictated by the rate of acceleration [35]. A high acceleration rate requires a high degree of horizontal propulsion [36], therefore the change in acceleration is greater when performing a 180° turn at higher running velocities, thereby resulting in greater energy expenditure.

The extent of the angle turned has also been shown to alter the biomechanical properties in running; a 90° turn exerts a significantly higher vertical, braking and propelling force than a 45° turn [37]. It would therefore be postulated that greater angles would also be associated with further increases in directional forces and thus energy expenditure during walking. In accord with this hypothesis, a linear relationship has been suggested between angle and energy expenditure when walking at 6 km∙hr^-1^ [18]. However, the present study suggests a synergistic interaction between speed and angle, with the influence of increasing angle within a speed only evident at 4.5 km∙hr^-1^ and above. This discrepancy may be attributable to differences in the walking velocities, the specific techniques used to turn, stature or training status [32, 38]. Indeed, both stature and peak $\dot{V}{\text{O}}_{2}$, an indicator of aerobic fitness and training status, were significant predictors of energy expenditure in the present study. Hatamoto et al. [16] previously suggested that ball game players, who are likely to be mainly running rather than walking and who turn more frequently anyway, were likely to have a more efficient turning technique. However, the mean $\dot{V}{\text{O}}_{2}$ of an individual turn was reported to be 0.34 ± 0.13 ml∙kg^-1^ and 0.55 ± 0.09 ml∙kg^-1^ at 4.3 km∙hr^-1^ and 5.4 km∙hr^-1^, respectively [16]. These values are substantially more than the values observed in the present study (Fig 2: 4.5 km∙hr^-1^ = 0.07 ± 0.03 ml∙kg^-1^; 5.5 km∙hr^-1^ = 0.13 ± 0.07 ml∙kg^-1^), despite the less trained status of the present participants. The reason for this discrepancy and its contradiction to the postulated role of aerobic fitness and technique are presently unclear, although it is perhaps pertinent to note the different methods of calculating the energy expenditure of an individual turn and the recent findings of Zagatto et al. [39] who found a lower metabolic power to be associated with more frequent changes of direction.

It is interesting to note the apparent dissociation between *VeDBA* and turning angle in the present study, whereby increasing the angle of the turn was not associated with any significant increase in *VeDBA*. This could be attributable to the short duration of the turns, although the high measurement resolution makes this unlikely, or measurement error associated with the use of magnetometry to isolate the turn. However, whilst the magnitude of change in the signal was decreased at lower turn angles, this is unlikely to entirely explain the present findings. Rather, this finding may largely be attributable to the complex and individual-specific interaction between the surge, heave and sway components of *DBA* as well as muscular effort that involves generation of high forces without the dynamism typical of straight-line travel. Indeed, recent studies using force plates to investigate turn kinetics suggest that during a turn, the surge (inline) component of *DBA* is accompanied by a sway (perpendicular) component (Griffiths et al., In press). Furthermore, the surge component tends to ‘average’ zero over the straight sections (equal deceleration and acceleration phases) but during a turn section, the surge component becomes negative on average to provide the deceleration required to enter and execute the turn. In addition, the heave component (vertical) component of *DBA* may increase above and beyond normative walking values but this may depend on the turning technique being employed, e.g. some participants may elect to turn using a ‘stop and reverse direction’ method while others may prefer a ‘gradual cornering’ approach. The authors are of the opinion that this is by far the most likely explanation for the lack of sensitivity of *VeDBA* to turns.

The present findings have significant implications within both sporting and health contexts given that few sporting, fitness or functional activities occur in a strictly linear fashion [37]. Indeed, whilst the present study only considered walking and caution should be exercised when extrapolating the findings to speeds associated with running and team sports, it is perhaps pertinent to note the similarity between the current findings and those reported elsewhere. Specifically, Dellal et al. [31] reported a greater heart rate, blood lactate and ratings of perceived exertion (RPE) during intermittent shuttle runs involving 180° turns compared to straight line running at the same average running velocities, subsequently confirmed by Buchheit et al. [32]. Furthermore, Bekraoui et al. [33] found that covering the same distance at the same average speed resulted in a significantly greater physiological response when the course was 3.5m compared to 7.0m. These earlier findings were recently extended by Hatamoto et al. [17] who found that, even at running speeds as low as 3 km∙hr^-1^, thirty 180° turns per minute elicited a similar metabolic demand as straight line running at 6 km∙hr^-1^. In the present study, a significant increase in total energy expenditure relative to straight line walking was not observed at 2.5 km∙hr^-1^, but was observed at 3.5 km∙hr^-1^. Whilst these findings are largely in accord with those of Hatamoto et al. [17], certain methodological differences should be considered, such as the training status of the sample population and turning frequencies utilised, which limit inter-study comparisons. Specifically, there were considerable differences in the number of turns completed, with Hatamoto et al. [17] utilising up to 30 turns per minute compared to the 35 turns in 3 minutes at 3.5 km∙hr^-1^ used in the present study. However, not all studies have found a significant influence of turning on energy expenditure, with Zamparo et al. [40] reporting no change in $\dot{V}{\text{O}}_{2}$ with increasing turn angle from 0 to 180°. This discrepancy may be related to the use of maximal running velocity during this study, thereby minimising the potential for further increases in $\dot{V}{\text{O}}_{2}$ to be elicited with increasing turn angle. Nonetheless, we would concur with Hatamoto et al. [16] that the energy expenditure associated with turning should be considered when estimating total energy expenditure during a football game in which more than 700 turns are typically completed per match [19].

From a health perspective, one important application of the present findings is in the design and interpretation of physical activity interventions. For example, the majority of energy expenditure prediction algorithms based on accelerometry data are derived from treadmill exercise. Such linear modes of locomotion are not cognisant of the additional metabolic costs associated with turning and this may, to some extent, contribute to the poor accuracy associated with the derived models during free-living conditions [41, 42]. Such inaccuracies are likely to be emphasised in certain populations, such as children, who are characterised by highly sporadic movements [43, 44]. Furthermore, accounting for the energy expenditure of turning could also be important in the evaluation of clinical trial effectiveness. Whilst the six-minute walking test is designed to be conducted over a 30m, straight line course with a 180° turn [20], reported distances covered range from 20 to 50 m [21, 22] due to space and resource limitations. Such discrepancies, using reference values reported by Chetta et al. [45] could result in the number of turns ranging from 12 to 32, which, according to the present data, may be associated with an additional $\dot{V}{\text{O}}_{2}$ expenditure ranging from 118 ml∙min^-1^ to 296 ml∙min^-1^. Swank et al. [46] demonstrated that a 6% improvement in peak $\dot{V}{\text{O}}_{2}$ was associated with a 5% decrease in risk of all-cause mortality in Congestive Heart Failure patients. Given the significantly lower peak aerobic capacity in patients, discrepancies arisen from failing to account for the energy expenditure of turning, which could be as much as 20% of a patients peak $\dot{V}{\text{O}}_{2}$, would considerably alter the interpretation of intervention efficacy. Future studies should seek to generate algorithms that account for distance and turns completed during a six-minute walk test, facilitating standardisation between centres.

There are certain limitations associated with the current study that should be acknowledged, such as the walking velocities utilised. Previous studies have employed higher running speeds, whereas we employed speeds more typical of habitual physical activity. Whilst this increased the generalisability of our findings to health contexts, caution should be taken when extrapolating these findings to a sporting context. Furthermore, although a strength of the study to optimise interpretation of our results, the controlled nature of the protocol limits ecological validity. Finally, although the walking speeds were associated with a moderate intensity of exercise for most of the participants, some may not have achieved a steady state $\dot{V}{\text{O}}_{2}$ within the 3-minute bout, thereby influencing the mean $\dot{V}{\text{O}}_{2}$ observed.

In conclusion, the present study demonstrated a synergistic interaction between speed and angle in determining the energy expenditure associated with walking. Specifically, 90° and 180° turns are associated with significant additional metabolic costs at 4.5 km∙hr^-1^ and above. These findings therefore highlight the importance of accounting for the quantity and magnitude of turns completed when estimating energy expenditure and have significant implications within both sport and health contexts.

## Supporting information

S1 File Minimum required dataset. (XLSX)

## References

[1] Singh GM, Danaei G, Farzadfar F, Stevens GA, Woodward M, Wormser D, et al The Age-Specific Quantitative Effects of Metabolic Risk Factors on Cardiovascular Diseases and Diabetes: A Pooled Analysis. PLoS One. 2013;8(7):e65174 doi: 10.1371/journal.pone.0065174 23935815 10.1371/journal.pone.0065174 PMC3728292

[2] Berrington de Gonzalez A, Hartge P, Cerhan JR, Flint AJ, Hannan L, MacInnis RJ, et al Body-Mass Index and Mortality among 1.46 Million White Adults. New Engl J Med. 2010;363(23):2211–9. doi: 10.1056/NEJMoa1000367 21121834 10.1056/NEJMoa1000367 PMC3066051

[3] Zheng W, McLerran DF, Rolland B, Zhang X, Inoue M, Matsuo K, et al Association between Body-Mass Index and Risk of Death in More Than 1 Million Asians. New Engl J Med. 2011;364(8):719–29. doi: 10.1056/NEJMoa1010679 21345101 10.1056/NEJMoa1010679 PMC4008249

[4] The Emerging Risk Factors C. Separate and combined associations of body-mass index and abdominal adiposity with cardiovascular disease: collaborative analysis of 58 prospective studies. The Lancet.377(9771):1085–95. 10.1016/S0140-6736(11)60105-0 PMC3145074 21397319

[5] Prospective Studies C. Body-mass index and cause-specific mortality in 900 000 adults: collaborative analyses of 57 prospective studies. The Lancet. 373(9669):1083–96. 10.1016/S0140-6736(09)60318-4 PMC2662372 19299006

[6] Effects) TGBoMRFfCDCBM. Metabolic mediators of the effects of body-mass index, overweight, and obesity on coronary heart disease and stroke: a pooled analysis of 97 prospective cohorts with 1·8 million participants. The Lancet.383(9921):970–83. 10.1016/S0140-6736(13)61836-X PMC3959199 24269108

[7] WHO. Child Health 2010.

[8] Kontis V, Mathers CD, Rehm J, Stevens GA, Shield KD, Bonita R, et al Contribution of six risk factors to achieving the 25×25 non-communicable disease mortality reduction target: a modelling study. The Lancet.384(9941):427–37. 10.1016/S0140-6736(14)60616-4 24797573

[9] WHO. Global action plan for the prevention and control of noncommunicable diseases 2013–2020. Geneva, Switzerland: 2013.

[10] Jakicic JM, Otto AD. Physical activity considerations for the treatment and prevention of obesity. Am J Clin Nutr. 2005;82(1):226S–9S. 16002826 10.1093/ajcn/82.1.226S

[11] Browning RC, Kram R. Energetic Cost and Preferred Speed of Walking in Obese vs. Normal Weight Women. Obesity Res. 2005;13(5):891–9. 10.1038/oby.2005.103 15919843

[12] Hagan RD, Upton SJ, Wong LES, Whittam J. The effects of aerobic conditioning and/or caloric restriction in overweight men and women. Med Sci Sports Exerc. 1986;18(1):87–94. 3457234

[13] Hill JO, Peters JC. Environmental Contributions to the Obesity Epidemic. Sci. 1998;280(5368):1371–4. 10.1126/science.280.5368.1371 9603719

[14] Jakicic JM, Winters C, Lang W, Wing RR. Effects of intermittent exercise and use of home exercise equipment on adherence, weight loss, and fitness in overweight women: A randomized trial. JAMA. 1999;282(16):1554–60. 10546695 10.1001/jama.282.16.1554

[15] Ludlow LW, Weyand PG. Energy expenditure during level human walking: seeking a simple and accurate predictive solution. JAPh. 2015. 10.1152/japplphysiol.00864.2015 26679617

[16] Hatamoto Y, Yamada Y, Fujii T, Higaki Y, Kiyonaga A, Tanaka H. A novel method for calculating the energy cost of turning during running. J Sports Med. 2013;4:117–22. 10.2147/OAJSM.S39206 PMC3871047 24379716

[17] Hatamoto Y, Yamada Y, Sagayama H, Higaki Y, Kiyonaga A, Tanaka H. The Relationship between Running Velocity and the Energy Cost of Turning during Running. PLoS One. 2014;9(1):e81850 doi: 10.1371/journal.pone.0081850 24497913 10.1371/journal.pone.0081850 PMC3908867

[18] Wilson RP, Griffiths IW, Legg PA, Friswell MI, Bidder OR, Halsey LG, et al Turn costs change the value of animal search paths. Ecol Lett. 2013;16(9):1145–50. doi: 10.1111/ele.12149 23848530 10.1111/ele.12149

[19] Bloomfield J, Polman R, O'Donoghue P. Physical Demands of Different Positions in FA Premier League Soccer. J Sports Sci Med. 2007;6(1):63–70. 24149226 PMC3778701

[20] ATS. ATS Statement: Guidelines for the Six-Minute Walk Test. Am J Respir Crit Care Med. 2002;166(1):111–7. doi: 10.1164/ajrccm.166.1.at1102 12091180 10.1164/ajrccm.166.1.at1102

[21] Lipkin DP, Scriven AJ, Crake T, Poole-Wilson PA. Six Minute Walking Test For Assessing Exercise Capacity In Chronic Heart Failure. Br Med J. 1986;292(6521):653–5. 3081210 10.1136/bmj.292.6521.653 PMC1339640

[22] Troosters T, Gosselink R, Decramer M. Six minute walking distance in healthy elderly subjects. Eur Respir J. 1999;14(2):270–4. 10515400 10.1034/j.1399-3003.1999.14b06.x

[23] Beekman E, Mesters I, Hendriks EJM, Klaassen MPM, Gosselink R, van Schayck OCP, et al Course length of 30 metres versus 10 metres has a significant influence on six-minute walk distance in patients with COPD: an experimental crossover study. J Physiother.59(3):169–76. doi: 10.1016/S1836-9553(13)70181-4 23896332 10.1016/S1836-9553(13)70181-4

[24] Ng SS, Tsang WW, Cheung TH, Chung JS, To FP, Yu PC. Walkway Length, But Not Turning Direction, Determines the Six-Minute Walk Test Distance in Individuals With Stroke. Arch Phys Med Rehabil. 2011;92(5):806–11. doi: 10.1016/j.apmr.2010.10.033 21530729 10.1016/j.apmr.2010.10.033

[25] Ng SS, Yu PC, To FP, Chung JS, Cheung TH. Effect of walkway length and turning direction on the distance covered in the 6-minute walk test among adults over 50 years of age: a cross-sectional study. Physiotherapy. 2013;99(1):63–70. doi: 10.1016/j.physio.2011.11.005 23219645 10.1016/j.physio.2011.11.005

[26] Jones AM, Doust JH. A 1% treadmill grade most accurately reflects the energetic cost of outdoor running. J Sports Sci. 1996;14(4):321–7. doi: 10.1080/02640419608727717 8887211 10.1080/02640419608727717

[27] Beaver WL, Wasserman K, Whipp BJ. A New Method of Detecting Anaerobic Threshold by Gas Exchange. JAPh. 1986;60:2020–7. 10.1152/jappl.1986.60.6.2020 3087938

[28] Shepard ELC, Wilson RP, Liebsch N, Quintana F, GÃƒÂ³mez Laich A, Lucke K. Flexible paddle sheds new light on speed: a novel method for the remote measurement of swim speed in aquatic animals. Endanger Spec Res. 2008;4(1–2):157–64.

[29] Gleiss AC, Wilson RP, Shepard ELC. Making overall dynamic body acceleration work: on the theory of acceleration as a proxy for energy expenditure. Methods Ecol Evol. 2011;2(1):23–33.

[30] Qasem L, Cardew A, Wilson A, Griffiths I, Halsey LG, Shepard ELC, et al Tri-Axial Dynamic Acceleration as a Proxy for Animal Energy Expenditure; Should We Be Summing Values or Calculating the Vector? PLoS One. 2012;7(2):e31187 doi: 10.1371/journal.pone.0031187 22363576 10.1371/journal.pone.0031187 PMC3281952

[31] Dellal A, Keller D, Carling C, Chaouachi A, Wong DP, Chamari K. Physiologic Effects of Directional Changes in Intermittent Exercise in Soccer Players. J Strength Cond Res. 2010;24(12):3219–26. doi: 10.1519/JSC.0b013e3181b94a63 19996785 10.1519/JSC.0b013e3181b94a63

[32] Buchheit M, Haydar B, Hader K, Ufland P, Ahmaidi S. Assessing running economy during field running with changes of direction: application to 20 m shuttle runs. Int J Sports Physiol Perform. 2011;6(3):380–95. 21911863 10.1123/ijspp.6.3.380

[33] Bekraoui N, Fargeas-Gluck M-A, Léger L. Oxygen uptake and heart rate response of 6 standardized tennis drills. Appl Physiol Nutr Metab. 2012;37(5):982–9. doi: 10.1139/h2012-082 22871149 10.1139/h2012-082

[34] Sheppard JM, Young WB. Agility literature review: Classifications, training and testing. J Sports Sci. 2006;24(9):919–32. doi: 10.1080/02640410500457109 16882626 10.1080/02640410500457109

[35] di Prampero PE, Fusi S, Sepulcri L, Morin JB, Belli A, Antonutto G. Sprint running: a new energetic approach. J Exp Biol. 2005;208(14):2809–16. 16000549 10.1242/jeb.01700

[36] Hunter JP, Marshall RN, McNair PJ. Relationships between Ground Reaction Force Impulse and Kinematics of Sprint-Running Acceleration. J Appl Biomech. 2005;21(1):31–43. 16131703 10.1123/jab.21.1.31

[37] Schot P, Dart J, Schuh M. Biomechanical Analysis of Two Change-of-Direction Maneuvers While Running. J Orthop Sports Phys Ther. 1995;22(6):254–8. doi: 10.2519/jospt.1995.22.6.254 8580952 10.2519/jospt.1995.22.6.254

[38] Zadro I, Sepulcri L, Lazzer S, Fregolent R, Zamparo P. A Protocol of Intermittent Exercise (Shuttle Runs) to Train Young Basketball Players. J Strength Cond Res. 2011;25(6):1767–73. doi: 10.1519/JSC.0b013e3181da85d1 21358430 10.1519/JSC.0b013e3181da85d1

[39] Zagatto AM, Ardigo LP, Barbieri FA, Milioni F, Iacono AD, Camargo BHF, et al Performance and metabolic demand of a new repeated-sprint ability test in basketball players: does the number of changes of direction matter? J Strength Cond Res. 2017. 10.1519/JSC.0000000000001710 28211843

[40] Zamparo P, Zadro I, Lazzer S, Beato M, Sepulcri L. Energetics of Shuttle Runs: The Effects of Distance and Change of Direction. Int J Sports Physiol Perform. 2014;9(6):1033–9. doi: 10.1123/ijspp.2013-0258 24700201 10.1123/ijspp.2013-0258

[41] Hendelman D, Miller K, Bagget C, Debold E, Freedson PS. Validiity of accelerometry for the assessment of moderate intensity physical activity in the field. Med Sci Sports Exerc. 2000;32:S442–S9. 10993413 10.1097/00005768-200009001-00002

[42] Swartz AM, Strath SJ, Bassett DR Jr., O'Brien WL, King GA, Ainsworth BE. Estimation of energy expenditure using CSA accelerometers at hip and wrist sites. Med Sci Sports Exerc. 2000;32(9 Suppl):S450–6. 10993414 10.1097/00005768-200009001-00003

[43] Bailey RB, Olson J, Pepper SL, Porszasz J, Barstow TJ, Cooper DM. The level and tempo of children's physical activities: an observational study. Med Sci Sports Exerc. 1995;27(7):1033–41. 7564970 10.1249/00005768-199507000-00012

[44] Baquet G, Stratton G, Van Praagh E, Berthoin S. Improving physical activity assessment in prepubertal children with high-frequency accelerometry monitoring: A methodological issue. Prev Med. 2007;44(2):143–7. doi: 10.1016/j.ypmed.2006.10.004 17157370 10.1016/j.ypmed.2006.10.004

[45] Chetta A, Zanini A, Pisi G, Aiello M, Tzani P, Neri M, et al Reference values for the 6-min walk test in healthy subjects 20–50 years old. Respir Med. 2006;100(9):1573–8. doi: 10.1016/j.rmed.2006.01.001 16466676 10.1016/j.rmed.2006.01.001

[46] Swank AM, Horton J, Fleg JL, Fonarow GC, Keteyian S, Goldberg L, et al Modest Increase in Peak VO_2_ Is Related to Better Clinical Outcomes in Chronic Heart Failure Patients: Results From Heart Failure and a Controlled Trial to Investigate Outcomes of Exercise Training. Circ Heart Fail. 2012;5(5):579–85. doi: 10.1161/CIRCHEARTFAILURE.111.965186 22773109 10.1161/CIRCHEARTFAILURE.111.965186 PMC3732187

